# Diagnostic Performance of 99mTc-MAG3 Renal Scintigraphy for Acute Tubular Necrosis and Acute Rejection in Kidney Transplant Recipients with Suspected Graft Dysfunction During the First 90 Days: A Single-Center, Biopsy-Referenced Retrospective Study

**DOI:** 10.3390/diagnostics16193193

**Published:** 2026-10-01

**Authors:** Ashraf Imam, Suha Shabaneh, Riham Imam, Michael Rivin, Ahmad Salhab, Itamar Tamir, Keren Tzukert, Roy Abel, Simona Ben-Haim, Harbi Khalayleh, Oded Cohen-Arazi, Abed Khalaileh

**Affiliations:** 1 Organ Transplantation Unit, Department of Surgery, Hadassah-Hebrew University of Jerusalem, Jerusalem 91120, Israel; suhasuha1987@gmail.com (S.S.); rivin@hadassah.org.il (M.R.); ahmad.salhab@mail.huji.ac.il (A.S.); caoded@hadassah.org.il (O.C.-A.); 2 Department of Radiology, Hadassah-Hebrew University of Jerusalem, Jerusalem 91120, Israel; imam1@hadassah.org.il (R.I.); itamart@hadassah.org.il (I.T.); 3 Department of Nephrology, Hadassah-Hebrew University of Jerusalem, Jerusalem 91120, Israel; kerentz@hadassah.org.il (K.T.); roy@hadassah.org.il (R.A.); 4 Department of Medical Biophysics and Nuclear Medicine, Hadassah-Hebrew University of Jerusalem, Jerusalem 91120, Israel; simonabu@hadassah.org.il; 5 Department of Surgery, Kaplan-Hebrew University of Jerusalem, Jerusalem 91120, Israel; harbikh@clalit.org.il

**Keywords:** 99mTc-MAG3, renal scintigraphy, kidney transplantation, acute tubular necrosis, delayed graft function, acute rejection, diagnostic accuracy, verification bias

## Abstract

**Background:** Acute tubular necrosis (ATN) and acute rejection are the principal parenchymal causes of early kidney allograft dysfunction, and biopsy is the reference standard for distinguishing them. We assessed 99mTc-MAG3 scintigraphy against biopsy. **Methods:** Single-center retrospective study of transplants (2016 to 2025) scanned within 90 days for suspected graft dysfunction: 42 transplants in 41 recipients, 10% of 420 screened. Accuracy was calculated only in the 25 with a paired biopsy, with exact 95% confidence intervals (CIs). Because biopsy was performed on clinical indication and not in all patients, verification bias was quantified by Begg–Greenes correction and extreme-case bounds. **Results:** Scintigraphy was performed a median of 8 days after transplantation; 69% had delayed graft function. Scans were read as ATN in 31 (74%), normal in seven (17%), rejection in three (7%) and obstruction in one; biopsy showed ATN in 15, rejection in six and normal histology in four. For ATN, sensitivity was 14/15 (93.3%; CI 68.1 to 99.8), specificity 7/10 (70.0%; 34.8 to 93.3) and positive predictive value 14/17 (82.4%; 56.6 to 96.2). For rejection, sensitivity was 3/6 (50.0%; 11.8 to 88.2) and specificity 19/19 (100%; 82.4 to 100), the predictive value of three positive scans being uninformative (CI 29.2 to 100). Correcting for verification placed ATN specificity between 29.2% and 76.9% and rejection sensitivity between 13.0% and 50.0%. **Conclusions:** Scintigraphy rarely missed biopsy-proven ATN but was not specific and identified only half of the rejection episodes. These estimates rest on small numbers in a clinically selected population; biopsy remains necessary whenever rejection is suspected, and they do not apply to routine or protocol scintigraphy.

## 1. Introduction

Kidney transplantation is the treatment of choice for most patients with end-stage kidney disease, offering better survival and quality of life at lower cost than long-term dialysis [1,2]. In the first weeks after transplantation, graft dysfunction is common and its two principal parenchymal causes, ischemic acute tubular necrosis (ATN), the usual substrate of delayed graft function (DGF), and acute rejection, present similarly but require opposite management: supportive care in the first case and intensified immunosuppression in the second [3,4,5].

ATN after transplantation is the histological expression of ischemia–reperfusion injury and is favored by donor factors, prolonged cold and warm ischemia, perioperative hypotension and, less often, early calcineurin-inhibitor toxicity [3,4,5,6,7]. It is the most frequent cause of DGF after deceased donor transplantation and is uncommon after living donor transplantation [8,9,10]. Acute rejection, whether T-cell-mediated or antibody-mediated, can develop from the first days after transplantation, is most frequent during the first months, and is classified histologically according to the Banff criteria [5,11]. The KDIGO clinical practice guideline for the care of kidney transplant recipients recommends close monitoring of serum creatinine and urine output, allograft ultrasonography as part of the assessment of dysfunction, and allograft biopsy when there is a persistent unexplained rise in creatinine, every 7 to 10 days during DGF, and when expected function is not achieved within 1 to 2 months [5]. Biopsy is the reference standard, but it is invasive, subject to sampling error and inter-observer variability, and carries a small but measurable risk of bleeding [5,12,13].

Dynamic renal scintigraphy with 99mTc-mercaptoacetyltriglycine (99mTc-MAG3) provides a non-invasive assessment of graft perfusion, tubular uptake and excretion, and yields interpretable images even when graft function is severely reduced [9,14,15,16,17,18]. A transplant perfusion index derived from the first-pass curves was introduced by Hilson and colleagues in 1978, with values below 150 considered normal and higher values indicating reduced perfusion, as seen in rejection and vascular complications [19]. Classical descriptions distinguish an ATN pattern, in which perfusion is preserved but parenchymal accumulation is progressive and excretion absent or delayed, from a rejection pattern, in which perfusion itself is reduced [9,20,21]. The overlap between these patterns and other causes of dysfunction is, however, well recognized, and the value of scintigraphy relative to Doppler ultrasonography and biopsy in contemporary practice is uncertain [12,22,23,24].

The aim of this study was to estimate the diagnostic performance of 99mTc-MAG3 scintigraphy, as interpreted in routine clinical practice, for the detection of biopsy-proven ATN and of biopsy-proven acute rejection in kidney transplant recipients investigated for suspected graft dysfunction during the first 90 days after transplantation, and to describe, on an exploratory basis, how scan findings and performance varied with donor type and with the timing of the scan.

## 2. Methods

### 2.1. Design and Settings

This is a single-center retrospective diagnostic-accuracy study using data from the prospectively maintained kidney transplant registry of Hadassah Medical Center, Jerusalem, reported in accordance with the STARD 2015 statement [25]. All kidney transplants performed between January 2016 and February 2025 were screened. During the study period all recipients received rabbit anti-thymocyte globulin induction intraoperatively together with intravenous methylprednisolone; tacrolimus and mycophenolate were added on the first postoperative day and continued with corticosteroids as maintenance immunosuppression. Induction was therefore uniform across the cohort, and neither the induction nor the maintenance protocol changed during the study period. Doppler ultrasonography of the allograft is the first-line examination for postoperative graft dysfunction at our center, in line with KDIGO recommendation 8.4 [5]. 99mTc-MAG3 scintigraphy is not a protocol examination; it is requested at the discretion of the treating team when dysfunction persists after vascular and urological causes have been excluded, or when the team wishes to characterize graft perfusion and parenchymal function before deciding on biopsy.

### 2.2. Eligibility Criteria and Indications for Scintigraphy

Transplants were eligible if 99mTc-MAG3 scintigraphy (Technescan MAG3™, Curium US LLC, Maryland Heights, MO, USA) was performed within 90 days of transplantation for one or more of the following indications: DGF, defined as the need for dialysis within the first 7 postoperative days, or oliguria; serum creatinine failing to fall, or rising by 20% to 25% or more above the post-transplant nadir; or persistent graft dysfunction after Doppler ultrasonography had excluded vascular and urological complications. Recipients were 18 years of age or older. Scans performed more than 90 days after transplantation, scans performed for indications unrelated to suspected dysfunction, and transplants with incomplete clinical, imaging or biopsy records were excluded. When more than one scan-biopsy episode occurred after the same transplant, only the first (index) episode was analyzed. A recipient who received two separate transplants during the study period contributed one index episode per transplant.

### 2.3. Population and Sampling

**Figure 1:**
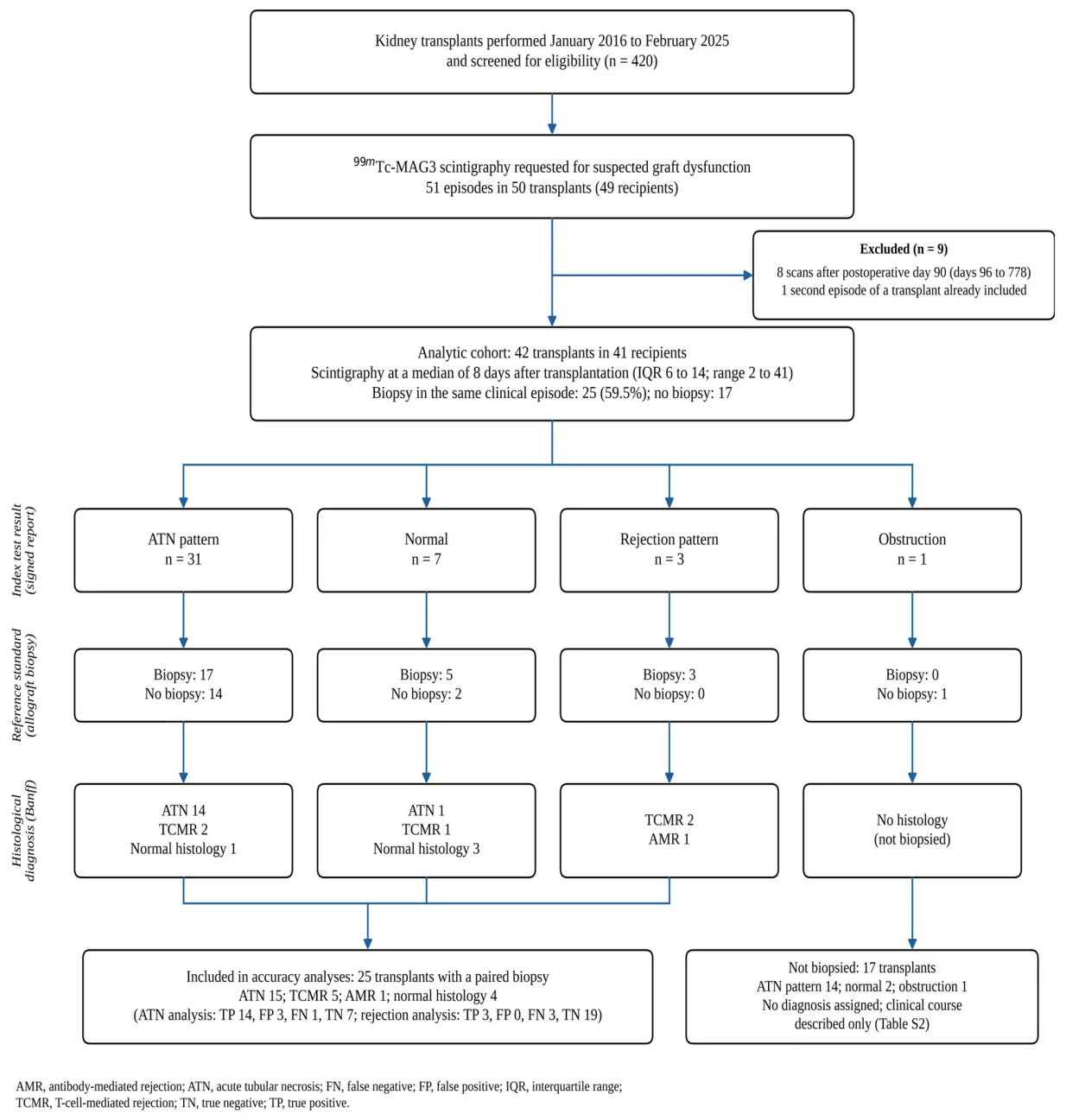
Participant flow (STARD 2015 diagram). Of 420 kidney transplants screened, ^99^ᵐTc-MAG3 scintigraphy was requested for suspected graft dysfunction in 51 episodes; 9 were excluded, leaving 42 transplants in 41 recipients. The index test result is the category recorded in the signed scintigraphy report; the reference standard is allograft biopsy performed during the same clinical episode. Diagnostic accuracy was calculated only in the 25 transplants with a paired biopsy; the 17 transplants without a biopsy were not assigned a diagnosis and are described in Supplementary Table S2. AMR = antibody-mediated rejection; ATN = acute tubular necrosis; FN = false negative; FP = false positive; IQR = interquartile range; TCMR = T-cell-mediated rejection; TN = true negative; TP = true positive.

The center registry contained 483 kidney transplants performed between January 2016 and February 2025; 63 were not screened because the medical record was incomplete, leaving 420 transplants for screening. Scintigraphy was requested for suspected graft dysfunction in 51 episodes (50 transplants in 49 recipients). Nine episodes were excluded: eight scans performed after day 90 (postoperative days 96 to 778) and one second episode after a transplant whose index episode was already included. The analytic cohort therefore comprised 42 transplants in 41 recipients (10.0% of transplants screened), of which 25 (59.5%) had an allograft biopsy during the same clinical episode (Figure 1). During the same period the center registry recorded delayed graft function in 83 of 469 transplants with a recorded status (17.7%), of whom 38 (46%) had a nuclear medicine study, confirming that scintigraphy was requested selectively rather than in all recipients with delayed graft function. A comparison of transplants that did and did not undergo scintigraphy is given in Supplementary Table S4.

### 2.4. Reference Standard, Definitions and Data Collection

The reference standard was percutaneous allograft biopsy, performed on clinical indication according to the KDIGO 2009 recommendations (persistent unexplained increase in serum creatinine; biopsy every 7 to 10 days during DGF; failure to achieve expected function within 1 to 2 months; new-onset proteinuria) [5] and read by the institutional renal pathologists according to the Banff classification current at the time [11]. Reporting pathologists did not have access to the scintigraphy report. Histological categories were ATN, T-cell-mediated rejection (TCMR), antibody-mediated rejection (AMR), normal histology and other. A biopsy was paired with the index scan if it was performed during the same clinical episode of dysfunction, before or after the scan; the interval between the two procedures is reported. For the accuracy analyses, ATN-positive was defined as histological ATN versus all other histological categories, and rejection-positive as TCMR or AMR versus all other categories. The scintigraphic index test result was the category recorded in the original signed report (ATN, acute rejection, normal, obstruction or other), dichotomized as ATN versus not ATN and as rejection versus not rejection. Transplants without a biopsy were not assigned a diagnosis and were not used to calculate any accuracy index; their scan category and clinical course are described only.

Clinical data were extracted from the electronic medical record: recipient age and sex, donor type (living; deceased after brain death, DBD; deceased after circulatory death, DCD), transplant number, pre-transplant dialysis modality, pre-formed donor-specific antibodies (DSAs), DGF, Doppler ultrasonography findings and maximal intrarenal resistive index, serum creatinine at the time of scintigraphy and on postoperative days 1, 30 and 90, and estimated glomerular filtration rate (eGFR) on days 1, 30 and 90 the CKD-EPI 2009 creatinine equation without the race coefficient. Cold ischemia time was retrieved from the transplant registry and is reported in Table 1; it was available for 24 of the 42 transplants.

### 2.5. Scintigraphy Acquisition and Interpretation

Studies were performed in the Department of Medical Biophysics and Nuclear Medicine according to the SNMMI/EANM practice guideline for renal scintigraphy in adults [23]. After intravenous bolus injection of 150 to 170 MBq of 99mTc-MAG3 with the patient supine and the gamma camera (Discovery NM/CT 670 (GE Healthcare, GE Medical Systems Israel, Tirat HaCarmel, Israel)) centered over the graft, a dynamic acquisition was obtained consisting of a perfusion phase (2 s frames for 60 s) followed by a function phase (10 s frames for 20 min). Regions of interest were drawn over the graft, the adjacent common or external iliac artery and a background area, and time–activity curves were generated. The Hilson transplant perfusion index was calculated from the first-pass curves of the graft and the iliac artery, with values below 150 considered normal [24].

Interpretation was qualitative, using the sequential images and the perfusion and renogram curves, with the perfusion index as a semi-quantitative adjunct. The categories used in the clinical reports were defined as follows. Normal: Prompt graft perfusion comparable to the iliac artery, prompt parenchymal uptake and timely excretion into the collecting system and bladder. ATN pattern: Preserved or only mildly reduced perfusion, progressive parenchymal accumulation and absent or markedly delayed excretion, giving a rising or plateau renogram curve. Rejection pattern: Reduced perfusion relative to the iliac artery (elevated perfusion index) with reduced parenchymal uptake and delayed excretion. Obstruction: Progressive retention of tracer in the collecting system with preserved parenchymal clearance. Other: Findings not fitting these categories, for example absent perfusion or urinary leak. Reports were issued as part of routine care by three board-certified nuclear medicine physicians, each with at least 10 years of experience in transplant renography, who had access to the clinical information (including donor type, dialysis requirement and laboratory results) and were not blinded. For this study the category recorded in the signed report was extracted without re-interpretation.

### 2.6. Ethical Considerations

The study was approved by the Institutional Review Board of Hadassah Medical Center (HMO-0168-25, 29 April 2025). The requirement for informed consent was waived because of the retrospective design and the use of anonymized data.

### 2.7. Statistical Analysis and Methods

Continuous variables are summarized as mean and standard deviation (SD) or as median and interquartile range (IQR) with range, and categorical variables as counts and percentages with the denominator stated whenever data were missing. Baseline characteristics of living and deceased donor recipients were compared descriptively with the Fisher exact test (Freeman–Halton extension for tables larger than 2 by 2, estimated by Monte Carlo simulation with 20,000 tables), the t test, or the Mann–Whitney test, as appropriate; these comparisons describe the two groups and are not tests of diagnostic performance.

Sensitivity, specificity, positive and negative predictive values and overall accuracy were calculated in transplants with a paired biopsy only, separately for ATN and for rejection, from 2 by 2 tables, with exact (Clopper–Pearson) 95% confidence intervals, and agreement between scintigraphy and histology was summarized with Cohen’s kappa. No pre-specified sample size was calculated; the sample comprised all eligible transplants, and the confidence intervals convey the precision achieved.

Because biopsy was performed on clinical indication and not in all patients, the accuracy estimates are subject to partial verification bias. We report the fraction of transplants verified by biopsy within each scan result, and we estimated verification-bias-corrected sensitivity and specificity with the Begg–Greenes method, which reweights verified cases by the inverse of the verification fraction in their scan-result stratum under the assumption that the decision to biopsy depended only on the scan result [26,27]. Confidence intervals for the corrected estimates were obtained by non-parametric bootstrap (5000 resamples of the 42 transplants). Because this assumption is unlikely to hold fully and correction methods perform poorly when false negatives are few [28], we also report extreme-case bounds obtained by assuming that all unverified transplants were classified either all correctly or all incorrectly by scintigraphy. As a third approach we defined, before re-analysis, a composite reference standard that was applied uniformly to all 42 transplants irrespective of the scan result. Where a biopsy existed, it took precedence. Where it did not, a transplant was classified as ATN-positive if it met all of three scan-independent criteria: dialysis-requiring delayed graft function in the first post-transplant week; no biopsy-proven or clinically treated rejection within 90 days; and recovery of graft function by day 90, defined as a serum creatinine below 221 µmol/L (2.5 mg/dL) or an estimated GFR of at least 30 mL/min/1.73 m^2^. A transplant was classified as rejection-positive if rejection was proven histologically or treated clinically within 90 days. No criterion used the scintigraphic result. Sensitivity analyses repeated the primary analysis restricted to scan-biopsy pairs no more than 30 days apart, retaining the two pairs for which the biopsy date was not recorded.

Analyses stratified by donor type, by the timing of the scan (within 14 days, 15 to 30 days, 31 to 90 days) and by era of transplantation (2016 to 2020, 2021 to 2025) are exploratory and are presented as counts with exact confidence intervals; no inferential comparison of accuracy between strata was performed, and no multivariable model was fitted, because the number of verified cases (25) and of events per outcome (at most 15 for ATN and 6 for rejection) precludes valid adjustment. Analyses were performed with SPSS version 23 (IBM Corp., Armonk, NY, USA) and Python 3.12 (SciPy, stats models).

## 3. Results

### 3.1. Patient Characteristics

The 42 transplants were performed in 41 recipients with a mean age of 51.6 ± 15.2 years (median 52; range 20 to 77); sex was recorded for 41 of the 42 transplants, of which 31 (76%) were in men. Twenty-two grafts (52%) came from living donors and 20 (48%) from deceased donors (8 DBD, 12 DCD). Deceased donor recipients were older, more often re-transplanted and far more often required dialysis in the first week (DGF in 18 of 20 versus 9 of 19 living donor recipients with data; *p* = 0.006; Table 1). Scintigraphy was performed at a median of 8 days after transplantation (IQR 6 to 14; range 2 to 41): 18 scans on days 2 to 7, 16 on days 8 to 14, six on days 15 to 30 and two on days 31 to 90, with no difference between donor groups (*p* = 0.68). Doppler ultrasonography preceded scintigraphy in all patients; findings were documented in the study database for 33 transplants, in which the median maximal intrarenal resistive index was 0.73 (IQR 0.66 to 0.77) and did not differ between donor groups (*p* = 0.58). One deceased donor graft had absented diastolic flow. Serum creatinine at the time of scintigraphy was a median of 4.1 mg/dL (IQR 2.8 to 6.3; *n* = 29 with a value recorded on the day of the scan).

### 3.2. Scintigraphic Findings and Their Relation to Clinical Presentation

**Figure 2:**
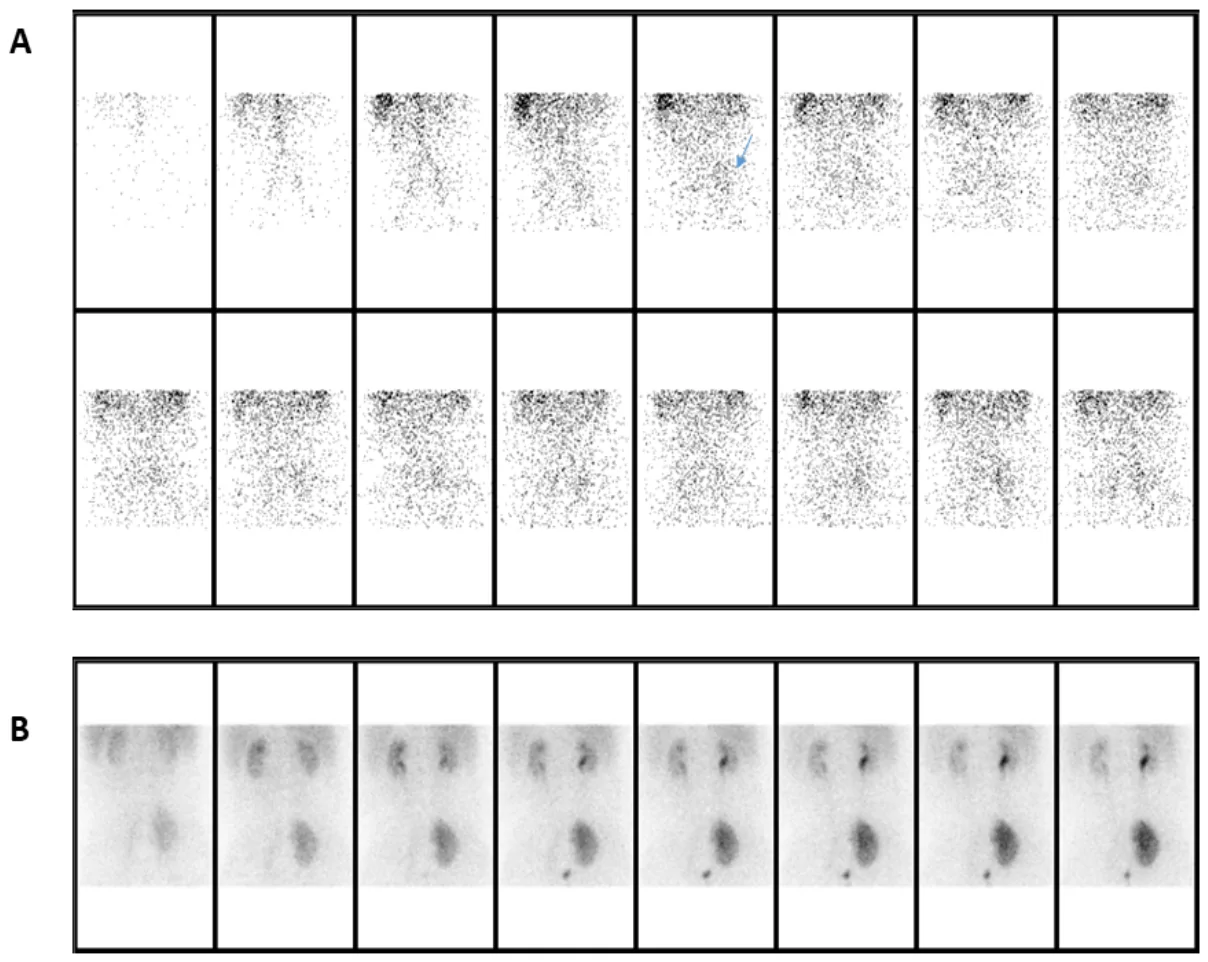
**ATN pattern.** A patient imaged with 99mTc-MAG3 on postoperative day 3 after living donor kidney transplantation. The graft in the left iliac fossa shows (**A**) preserved perfusion and (**B**) progressive parenchymal accumulation without significant excretion, consistent with an ATN pattern. Allograft biopsy on the following day showed acute tubular necrosis. Note faint uptake in the native kidneys.

**Figure 3:**
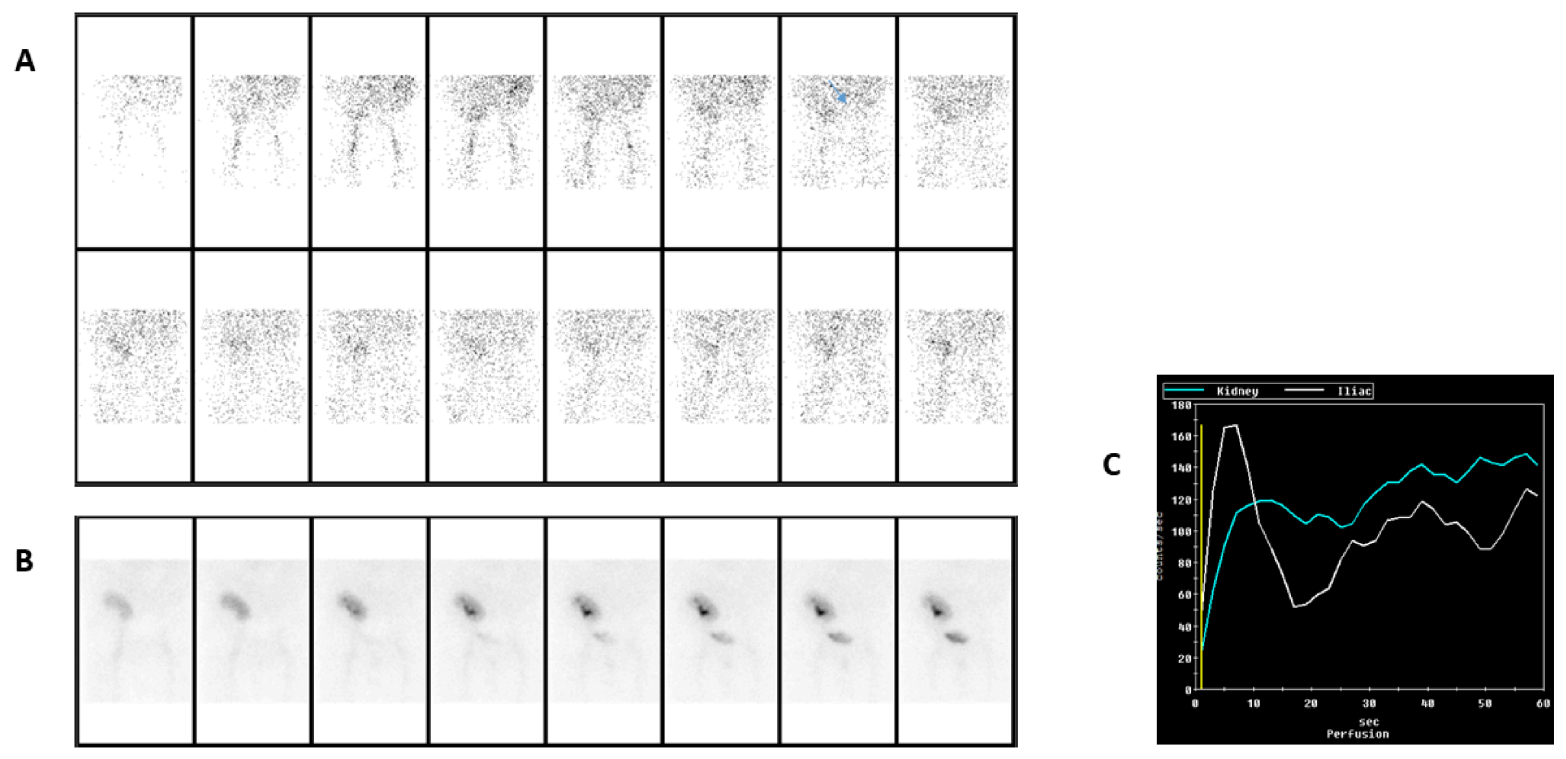
**Rejection pattern in a deceased donor kidney transplant recipient.** (**A**) Perfusion phase: Reduced graft perfusion relative to the iliac artery. (**B**) Function phase: Reduced parenchymal uptake with delayed excretion. (**C**) First-pass time–activity curves of the graft (green) and iliac artery (white), giving an elevated Hilson perfusion index. Allograft biopsy confirmed acute T-cell-mediated rejection. The yellow line indicates the time of radiotracer injection.

Scintigraphy was read as an ATN pattern in 31 transplants (73.8%), normal in seven (16.7%), a rejection pattern in three (7.1%) and obstruction in one (2.4%) (Figure 2 and Figure 3). The distribution differed between donor groups (Fisher–Freeman–Halton *p* = 0.003): all three rejection-pattern scans and the obstruction occurred in deceased donor recipients, whereas all seven normal scans occurred in living donor recipients (7 of 22 versus 0 of 20; *p* = 0.009) (Table 2). This difference paralleled the clinical presentation. Of the 27 recipients with DGF, 24 had an ATN-pattern scan and three a rejection-pattern scan; of the 12 without DGF, six had a normal scan, five an ATN pattern and one obstruction, and none had a rejection pattern. Serum creatinine on the day of the scan was a median of 5.7 mg/dL in recipients with an ATN-pattern scan and 1.4 mg/dL in those with a normal scan.

### 3.3. Biopsy Findings and the Relation Between Scintigraphy and Biopsy

Twenty-five transplants (59.5%) were biopsied during the same clinical episode: 11 of 20 deceased donor and 14 of 22 living donor grafts (Table 2). Histology showed ATN in 15 (60%), TCMR in five (20%), AMR in one (4%) and normal histology in four (16%). The 17 transplants that were not biopsied had scans read as ATN pattern in 14, normal in two and obstruction in one; their clinical course is summarized in Supplementary Table S2 and was not used as a reference standard. Biopsy dates were available for 23 of the 25 paired cases: the biopsy was performed a median of 1 day after the scan (IQR 2 days before to 6 days after; range 10 days before to 43 days after), preceded the scan in seven cases (30%), and was within 7 days of the scan in 16 cases and within 14 days in 21 cases. The single pair separated by more than 30 days (scan on day 41 read as rejection; AMR on biopsy 43 days later) was retained in the primary analysis and removed in a sensitivity analysis; the two pairs without a recorded biopsy date were retained, giving 24 pairs for that analysis. The full cross-tabulation of scintigraphy category against histology is shown in Table 3.

### 3.4. Diagnostic Performance for Acute Tubular Necrosis

Among the 25 biopsied transplants, scintigraphy identified 14 of the 15 with histological ATN (sensitivity 93.3%; 95% CI 68.1 to 99.8); the single false negative was a living donor graft with a normal scan on day 22 and ATN on a biopsy performed 9 days earlier. Of the 10 biopsied transplants without histological ATN, seven had a scan not read as ATN (specificity 70.0%; 95% CI 34.8 to 93.3); the three false positives were two TCMR and one normal biopsy after ATN-pattern scans. The positive predictive value was 14/17 (82.4%; 95% CI 56.6 to 96.2), the negative predictive value 7/8 (87.5%; 47.3 to 99.7), overall accuracy 21/25 (84.0%; 63.9 to 95.5) and kappa 0.66 (Table 4). The sensitivity estimates rests on a single false negative, and the width of the confidence intervals reflects the small number of verified cases.

Verification by biopsy occurred in 17 of 31 ATN-pattern scans (55%) and in 8 of 11 other scans (73%). Under the assumption that verification depended only on the scan result, the Begg–Greenes corrected sensitivity was 94.9% (bootstrap 95% CI 83.3 to 100) and the corrected specificity 63.8% (36.0 to 100). The extreme-case bounds, obtained by assigning all 17 unverified transplants either all correct or all incorrect classifications, were 77.8% to 96.6% for sensitivity and 29.2% to 76.9% for specificity (Table 4). Under the composite reference standard applied to all 42 transplants, which classified 7 of the 17 non-biopsied transplants as ATN-positive and 10 as ATN-negative, sensitivity was 21/22 (95.5%; 95% CI 77.2 to 99.9) but specificity fell to 10/20 (50.0%; 27.2 to 72.8) and the positive predictive value to 21/31 (67.7%; 48.6 to 83.3). All three approaches therefore agree that sensitivity for ATN is high and that the observed specificity of 70% is the most optimistic of the available estimates.

### 3.5. Diagnostic Performance for Acute Rejection

Six biopsied transplants had rejection (five TCMR, one AMR). Scintigraphy showed a rejection pattern in three of them (sensitivity 50.0%; 95% CI 11.8 to 88.2); the three rejections that were not recognized had scans read as ATN pattern in two (one deceased and one living donor graft) and as normal in one (living donor graft), and in the two with a recorded perfusion index the index was normal (44 and 31), that is, perfusion was preserved. None of the 19 biopsied transplants without rejection had a rejection-pattern scan (specificity 100%; 95% CI 82.4 to 100). The positive predictive value of 3/3 (100%) has a 95% confidence interval of 29.2 to 100 and is uninformative; the negative predictive value was 19/22 (86.4%; 65.1 to 97.1) and kappa 0.60 (Table 4). All three rejection-pattern scans were verified by biopsy, whereas 22 of 39 other scans (56%) were verified; the Begg–Greenes corrected sensitivity was 36.1% (bootstrap 95% CI 10.5 to 100) with unchanged specificity, and the extreme-case bounds for sensitivity were 13.0% to 50.0%. Under the composite reference standard, which identified no additional rejection episode among the non-biopsied transplants, sensitivity was unchanged at 3/6 (50.0%) with a specificity of 36/36 (100%; 90.3 to 100) and a negative predictive value of 36/39 (92.3%; 79.1 to 98.4).

### 3.6. Exploratory Analyses by Donor Type, Timing and Era

Stratum-specific counts are shown in Table 5 and Supplementary Table S3. For ATN, scintigraphy correctly classified 9 of 11 biopsied deceased donor grafts (sensitivity 6/6, specificity 3/5) and 12 of 14 living donor grafts (sensitivity 8/9, specificity 4/5). For rejection, three of four rejections in deceased donor grafts and zero of two in living donor grafts were recognized. Within scans performed in the first 14 days (*n* = 34; 20 biopsied), sensitivity for ATN was 13/13 and specificity 4/7, and sensitivity for rejection was 1/4 with specificity 16/16; among scans on days 15 to 30 (*n* = 6; 4 biopsied) sensitivity for ATN was 1/2 with specificity 2/2, and the single rejection was recognized. Only one biopsied transplant was scanned after day 30. Across eras, 11 of the 14 true-positive ATN scans occurred in transplants performed in 2016 to 2020, whereas all three rejection-pattern scans and five of the six biopsy-proven rejections occurred in 2021 to 2025. These strata are too small to support any inference and are reported for completeness.

## 4. Discussion

Radionuclide renography has been used to evaluate kidney allografts for almost five decades. Dynamic imaging with 99mTc-MAG3 provides, in a single study, an assessment of graft perfusion during the first pass of the tracer, of tubular extraction and of excretion, and the transplant perfusion index introduced by Hilson and colleagues quantifies perfusion relative to the adjacent artery, with values above 150 indicating reduced flow [19]. Early descriptions distinguished an ATN pattern, in which perfusion is preserved while extraction is progressive and excretion absent, from a rejection pattern, in which perfusion itself is reduced, and reported that the perfusion index together with the sequential images usually allowed the two to be separated [9,19,21]. Subsequent experience tempered this optimism. In a prospective series of 213 consecutive transplants imaged three times weekly, Heaf and Iversen found that the initial renogram was a useful marker of ischemic injury and predicted the duration of non-function and graft survival, but that serial scintigraphy did not contribute to the differential diagnosis between acute rejection, acute tubulointerstitial injury and calcineurin-inhibitor toxicity, and that its diagnostic specificity was insufficient for it to be the definitive investigation for the common diagnostic problems after transplantation [22,29,30]. Quantitative parameters, including the perfusion index, have shown only moderate interobserver agreement and overlapping distributions across histological categories [24]. In the largest contemporary series, a dual-center study of 377 recipients scanned within three days of transplantation, qualitative curve grading predicted DGF lasting 7 days or more with a sensitivity of 87% but a specificity of only 65% [31], and a second scan a few days later added little clinical information [32]. Reviews of post-transplant imaging therefore assign scintigraphy a limited and adjunctive role, mainly in characterizing the severity of parenchymal injury and in detecting vascular occlusion and urinary leak, while Doppler ultrasonography, which is inexpensive, performed at the bedside and free of radiation, has become the first line and in many units the only imaging modality [12,23,33,34,35]. Ultrasonography has its own limitations: the intrarenal resistive index, the most widely used Doppler parameter, was shown in a prospective study of 321 recipients with protocol biopsies to reflect recipient characteristics such as age and pulse pressure rather than graft histology, although it rises in the presence of graft dysfunction [36]. The KDIGO guideline accordingly recommends ultrasonography as part of the assessment of graft dysfunction and biopsy as the means of establishing its cause [5].

Against this background our findings are unsurprising and, for ATN, consistent with the older literature. In a selected cohort of recipients investigated for suspected dysfunction at a median of 8 days after transplantation, most of whom had DGF, an ATN-pattern scan identified 14 of 15 biopsy-proven ATN, with a specificity of 70% and a positive predictive value of 82%. The three false-positive ATN-pattern scans comprised two TCMR and one normal biopsy; the two rejections are the clinically important errors, because an ATN label in a patient with unrecognized rejection may delay treatment. The single false negative was a normal scan in a living donor recipient biopsied 9 days earlier. The verification-bias analysis suggests that the true sensitivity for ATN is probably high (corrected estimate 95%; extreme-case lower bound 78%) but that specificity may be considerably lower than observed (corrected estimate 64%; lower bound 29%), because many ATN-pattern scans in patients whose dysfunction resolved were never verified and some of these may have represented other pathology. A composite reference standard applied uniformly to all 42 transplants, using clinical criteria that do not involve the scan, gave the same picture in a different way: sensitivity 96% but specificity 50% and positive predictive value 68%. Three methods resting on different assumptions therefore converge on the conclusion that the observed specificity of 70% is the upper end of the plausible range rather than a point estimate. Our estimates for ATN are broadly comparable with the sensitivity of 87% and specificity of 65% reported for the prediction of prolonged DGF by curve grading [31], with the caveat that the endpoints differ: we used histology, whereas that study used the duration of DGF.

The low incidence of biopsy-proven rejection in this cohort (6 of 42 transplants) should be read in the light of a protocol in which every recipient received depleting anti-thymocyte globulin induction. Predictive values depend on prevalence, so the negative predictive value reported here would be lower in centers using non-depleting or risk-stratified induction, in which early rejection is more common. For rejection, the message of this study is negative and should be stated plainly. Only three scans in 42 were read as a rejection pattern; all three were confirmed, but half of the biopsy-proven rejections were not recognized, and after correction for verification bias the sensitivity is closer to one third. The confidence interval for the positive predictive value (29% to 100%) is too wide to support any claim that a rejection-pattern scan obviates biopsy. The two rejections read as ATN had preserved perfusion, which illustrates the physiological reason for the insensitivity of scintigraphy: T-cell-mediated rejection confined to the tubulointerstitium does not reduce first-pass perfusion, and the resulting curve is indistinguishable from that of ATN [21,22,24]. Rejection-pattern scans were confined to deceased donor recipients with DGF, and normal scans to living donor recipients without DGF. The different distribution of scan categories between donor groups therefore mirrors the different severity of dysfunction at the time of imaging rather than any donor-specific property of the test; the reporting physicians were, however, aware of donor type and clinical course, and an influence of clinical priors on interpretation cannot be excluded.

**Figure 4:**
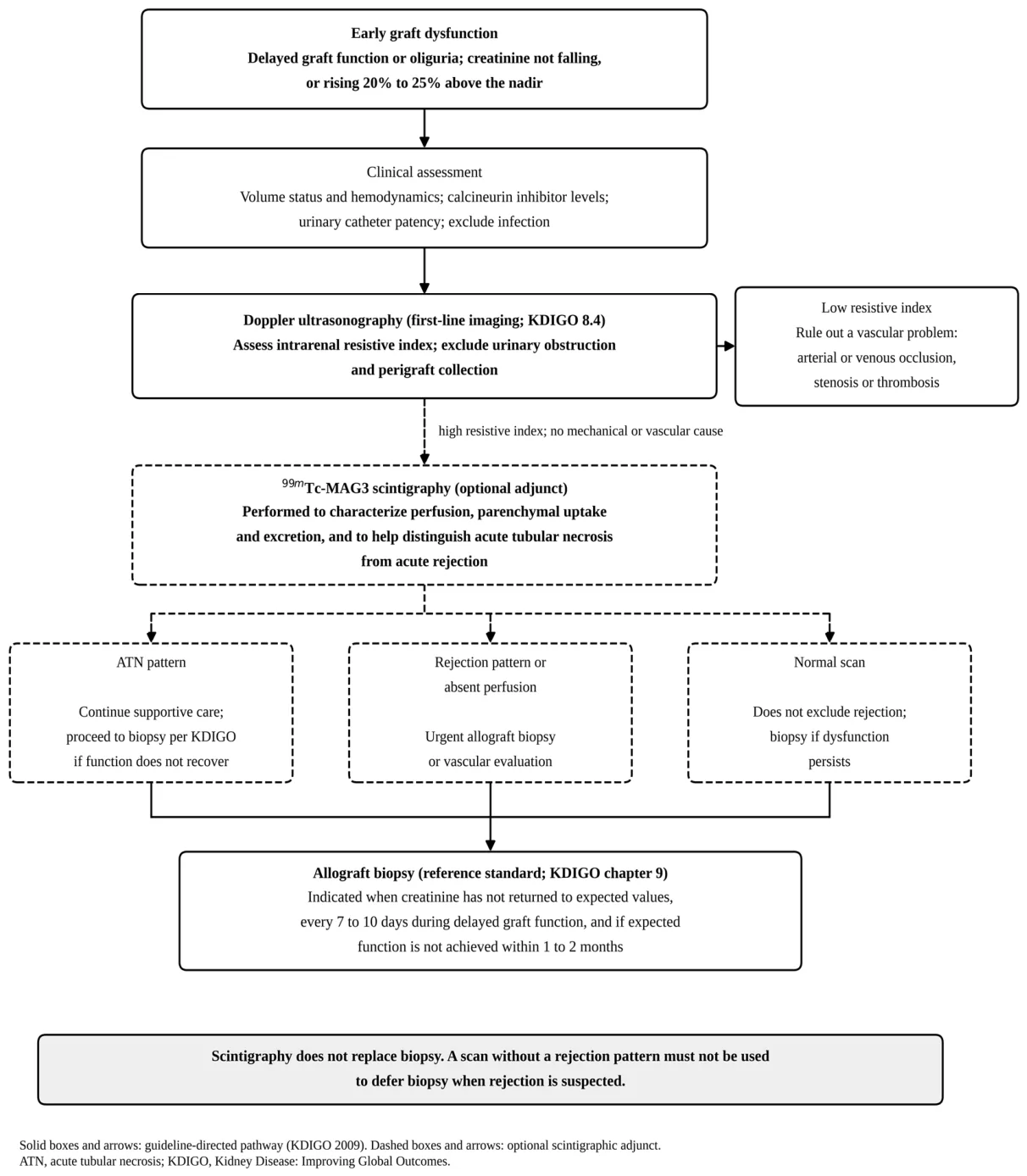
**Suggested place of 99mTc-MAG3 scintigraphy in the evaluation of early graft dysfunction, within the KDIGO 2009 framework** [5]. Solid boxes and arrows denote the guideline-directed pathway; dashed boxes and arrows denote the optional scintigraphic adjunct. ATN = acute tubular necrosis; KDIGO = kidney disease: Improving Global Outcomes.

The practical implication is modest and is illustrated in Figure 4, which follows the KDIGO framework [5]. In a unit with experience in transplant renography, scintigraphy may be considered after Doppler ultrasonography when dysfunction persists, to document severely impaired parenchymal function or absent perfusion and to support conservative management while a biopsy is arranged or in a patient in whom biopsy is temporarily contraindicated. It should not be used to defer biopsy in a patient in whom rejection is clinically suspected, because a scan without a rejection pattern does not exclude rejection, and a rejection-pattern scan requires histological confirmation before treatment. Whether the information provided by scintigraphy changes management or outcomes was not examined in this study and remains unproven.

This study has important limitations. First, biopsy was performed on clinical indication in 60% of transplants, and the decision to biopsy was not independent of the scan result. The resulting partial verification bias is expected to overestimate sensitivity and specificity when verification is more frequent after positive scans; in this cohort verification was in fact slightly more frequent after non-ATN scans, but every rejection-pattern scan and only 56% of other scans were verified, so rejections among unverified patients with non-rejection scans would have been missed, and the corrected sensitivity for rejection is lower than the observed value. We have reported corrected estimates, bounds and a composite reference standard, but the corrections rest on assumptions that cannot be tested in these data, and correction methods are unreliable when false negatives are few [26,28]. The composite standard is itself imperfect: its clinical criteria were recorded during care in which the scan was available to the treating team, so although no criterion uses the scan result, residual incorporation bias cannot be excluded, and a clinical course compatible with resolving ATN is not equivalent to histological ATN. Second, the number of verified cases (25) and of rejections (six) is small, so the confidence intervals are wide, and the donor type, timing and era strata are exploratory only. Third, the cohort is a clinically selected referral population, 10% of transplants performed in the study period, with a high pre-test probability of parenchymal injury (69% DGF); only registry-level data were available for transplants that were not scanned (Supplementary Table S4), so the estimates cannot be generalized to unselected recipients or to protocol scintigraphy. Fourth, scans were interpreted in routine practice by reporting physicians who were aware of the clinical information, including donor type, and no blinded second read was performed for this study, so reproducibility could not be assessed; cortical retention indices and transit times were not systematically recorded. Fifth, the biopsy itself is subject to sampling error and inter-observer variability, the interval between scan and biopsy was up to 43 days in one case, and the histological reference categories may have been influenced by the evolving Banff classification during the study period. Sixth, the study spans nine years during which immunosuppression and management practice may have changed, and cold ischemia time was available for only 24 transplants. Induction and maintenance immunosuppression, by contrast, were uniform throughout and therefore cannot account for any of the differences reported here. With the number of events observed, no multivariable adjustment would have been valid in any case. A further caveat applies to the renal function data: estimated GFR was derived from the CKD-EPI 2009 creatinine equation, which assumes a steady state and is not validated in the first days after transplantation or during dialysis-requiring delayed graft function. The day 1 values should therefore be read as a descriptive index of early graft function rather than as a measurement of glomerular filtration rate, and the day 30 and day 90 values are the more interpretable. A definitive evaluation would require a prospective multicenter design with scintigraphy at a fixed time point, systematic biopsy irrespective of scan result, standardized quantitative analysis and blinded reading by multiple observers.

## 5. Conclusions

In a single-center, clinically selected series of kidney transplant recipients investigated for suspected graft dysfunction in the first 90 days, an ATN pattern on 99mTc-MAG3 scintigraphy was sensitive for biopsy-proven ATN, whereas a rejection pattern was uncommon and insensitive: half of biopsy-proven rejections were not recognized. The estimates rest on small numbers and are subject to partial verification bias. Within these limits, scintigraphy interpreted by experienced readers may support, but cannot replace, clinical assessment, Doppler ultrasonography and allograft biopsy in the management of early graft dysfunction; a scan without a rejection pattern must not be used to defer biopsy when rejection is suspected. Prospective studies with systematic biopsy and blinded, standardized reading are needed before any role in a diagnostic algorithm can be recommended.

## Figures and Tables

**Table 1:** Characteristics of the study population by donor type.

Characteristic	Deceased Donor (*n* = 20)	Living Donor (*n* = 22)	Total (*n* = 42)	*p*
Recipient age, years, mean ± SD (range)	55.6 ± 14.8 (20–77)	48.0 ± 15.0 (22–76)	51.6 ± 15.2 (20–77)	0.10
Male sex, *n*/*N* (%)	17/20 (85)	14/21 (67)	31/41 (76)	0.28
Donor subtype, *n*	DBD 8; DCD 12	Living 22		
Retransplantation (second or third graft), *n* (%)	6 (30)	1 (5)	7 (17)	0.041
Pre-emptive transplantation, *n*/*N* (%)	1/19 (5)	5/19 (26)	6/38 (16)	0.18
Pre-formed donor-specific antibodies, *n*/*N* (%)	6/20 (30)	6/19 (32)	12/39 (31)	1.00
Delayed graft function (dialysis within 7 days), *n*/*N* (%)	18/20 (90)	9/19 (47)	27/39 (69)	0.006
Doppler ultrasonography documented, *n*	16	17	33	
Maximal resistive index, median (IQR)	0.71 (0.66–0.78) [*n* = 14]	0.73 (0.66–0.75) [*n* = 17]	0.73 (0.66–0.77) [*n* = 31]	0.58
Absent diastolic flow, *n*	1	0	1	
Postoperative day of scintigraphy, median (IQR; range)	8 (7–13; 2–41)	8.5 (5–14; 2–35)	8 (6–14; 2–41)	0.68
Scintigraphy within 14 days, *n* (%)	17 (85)	17 (77)	34 (81)	
Serum creatinine at scintigraphy, mg/dL, median (IQR)	4.8 (4.0–6.4) [*n* = 13]	3.4 (1.9–5.8) [*n* = 16]	4.1 (2.8–6.3) [*n* = 29]	
Allograft biopsy in the same episode, *n* (%)	11 (55)	14 (64)	25 (60)	0.75
Biopsy-proven rejection within 90 days, *n* (%)	4 (20)	2 (9)	6 (14)	0.40
Serum creatinine, mg/dL, mean ± SD				
Postoperative day 1	6.5 ± 1.7	5.2 ± 1.9	5.8 ± 1.9	
Postoperative day 30	3.9 ± 3.1 [*n* = 18]	2.2 ± 1.6	3.0 ± 2.5 [*n* = 40]	
Postoperative day 90	3.0 ± 2.2 [*n* = 18]	1.9 ± 1.2 [*n* = 21]	2.4 ± 1.8 [*n* = 39]	
Estimated GFR, mL/min/1.73 m^2^, mean ± SD				
Postoperative day 1	9.7 ± 4.0	14.0 ± 5.4	11.9 ± 5.2	
Postoperative day 30	26.6 ± 17.7 [*n* = 18]	52.2 ± 27.0	40.7 ± 26.3 [*n* = 40]	
Postoperative day 90	33.0 ± 17.4 [*n* = 18]	55.6 ± 27.6 [*n* = 21]	45.2 ± 25.8 [*n* = 39]	
Cold ischaemia time, hours, mean ± SD	10.5 ± 3.1 [*n* = 12]	3.4 ± 0.9 [*n* = 12]	6.9 ± 4.3 [*n* = 24]	<0.001

Values are *n* (%), mean ± SD (range) or median (IQR) as indicated; denominators are given where data were missing. *p* values compare deceased with living donor recipients (Fisher exact test, *t* test or Mann–Whitney test) and are descriptive. DBD = donation after brain death; DCD = donation after circulatory death; GFR = glomerular filtration rate; IQR = interquartile range; SD = standard deviation.

**Table 2:** Scintigraphy categories, biopsy performance and histological diagnoses by donor type.

Variable	Deceased Donor (*n* = 20)	Living Donor (*n* = 22)	Total (*n* = 42)	*p*
Scintigraphy category, *n*				0.003
ATN pattern	16	15	31	
Rejection pattern	3	0	3	
Normal	0	7	7	
Obstruction	1	0	1	
Delayed graft function among ATN-pattern scans, *n*/*N*	15/16	9/13	24/29	
Delayed graft function among normal scans, *n*/*N*	–	0/6	0/6	
Allograft biopsy performed, *n* (%)	11 (55)	14 (64)	25 (60)	0.75
Histological diagnosis among biopsied, *n*				0.61
ATN	6	9	15	
T-cell-mediated rejection	3	2	5	
Antibody-mediated rejection	1	0	1	
Normal histology	1	3	4	
Scintigraphy category among transplants not biopsied, *n*				
ATN pattern	8	6	14	
Normal	0	2	2	
Obstruction	1	0	1	

*p* values (Fisher exact test, with the Freeman–Halton extension for tables larger than 2 × 2) compare deceased with living donor recipients and are descriptive. ATN = acute tubular necrosis. Delayed graft function status was missing in three living donor transplants (two ATN-pattern scans and one normal scan).

**Table 3:** Cross-tabulation of scintigraphy category and histological diagnosis in the 25 transplants with a paired allograft biopsy.

Scintigraphy Category	ATN	T-Cell-Mediated Rejection	Antibody-Mediated Rejection	Normal Histology	Total
ATN pattern	14	2	0	1	17
Rejection pattern	0	2	1	0	3
Normal	1	1	0	3	5
Obstruction	0	0	0	0	0
Total	15	5	1	4	25

ATN = acute tubular necrosis. For the ATN analysis, true positives (14) are in the first cell of the first row and true negatives (7) in the non-ATN cells of the other rows; for the rejection analysis, true positives (3) are the rejection cells of the second row and true negatives (19) the non-rejection cells of the other rows.

**Table 4:** Diagnostic performance of ^99m^Tc-MAG3 scintigraphy for biopsy-proven acute tubular necrosis and acute rejection in the 25 transplants with a paired biopsy, with verification-bias analysis and sensitivity analyses. Confidence intervals are exact (Clopper–Pearson) unless stated.

Index	ATN (Reference: Histological ATN)	Acute Rejection (Reference: TCMR or AMR)
True positive/false positive/false negative/true negative	14/3/1/7	3/0/3/19
Sensitivity, *n*/*N*, % (95% CI)	14/15, 93.3 (68.1–99.8)	3/6, 50.0 (11.8–88.2)
Specificity, *n*/*N*, % (95% CI)	7/10, 70.0 (34.8–93.3)	19/19, 100 (82.4–100)
Positive predictive value, *n*/*N*, % (95% CI)	14/17, 82.4 (56.6–96.2)	3/3, 100 (29.2–100)
Negative predictive value, *n*/*N*, % (95% CI)	7/8, 87.5 (47.3–99.7)	19/22, 86.4 (65.1–97.1)
Accuracy, *n*/*N*, % (95% CI)	21/25, 84.0 (63.9–95.5)	22/25, 88.0 (68.8–97.5)
Cohen’s kappa	0.66	0.60
Verification fraction, scan positive/scan negative	17/31 (55%)/8/11 (73%)	3/3 (100%)/22/39 (56%)
Begg–Greenes corrected sensitivity, % (bootstrap 95% CI)	94.9 (83.3–100)	36.1 (10.5–100)
Begg–Greenes corrected specificity, % (bootstrap 95% CI)	63.8 (36.0–100)	100 (100–100)
Extreme-case bounds, sensitivity/specificity, %	77.8–96.6/29.2–76.9	13.0–50.0/100
Composite reference standard (all 42 transplants): TP/FP/FN/TN	21/10/1/10	3/0/3/36
Composite reference standard: sensitivity, % (95% CI)	95.5 (77.2–99.9) [21/22]	50.0 (11.8–88.2) [3/6]
Composite reference standard: specificity, % (95% CI)	50.0 (27.2–72.8) [10/20]	100 (90.3–100) [36/36]
Composite reference standard: PPV/NPV, % (95% CI)	67.7 (48.6–83.3)/90.9 (58.7–99.8)	100 (29.2–100)/92.3 (79.1–98.4)
Sensitivity analysis: biopsy within 30 days of scan (*n* = 24): sensitivity/specificity/PPV/NPV, %	93.3/66.7/82.4/85.7	40.0 (2/5)/100/100 (2/2)/86.4

AMR = antibody-mediated rejection; ATN = acute tubular necrosis; CI = confidence interval; NPV = negative predictive value; PPV = positive predictive value; TCMR = T-cell-mediated rejection. Extreme-case bounds assume that all transplants not verified by biopsy were classified either all correctly (upper bound) or all incorrectly (lower bound). The composite reference standard is defined in Section 2.7; it classified 7 of the 17 non-biopsied transplants as ATN-positive and none as rejection-positive, and uses no information derived from the scan.

**Table 5:** Exploratory stratification of diagnostic performance by donor type (transplants with a paired biopsy). Counts are given with exact 95% confidence intervals; no inferential comparison between strata was performed.

Index	Deceased Donor (*n* = 11 Biopsied)	Living Donor (*n* = 14 Biopsied)
ATN: TP/FP/FN/TN	6/2/0/3	8/1/1/4
ATN: Sensitivity, *n*/*N*, % (95% CI)	6/6, 100 (54.1–100)	8/9, 88.9 (51.8–99.7)
ATN: Specificity, *n*/*N*, % (95% CI)	3/5, 60.0 (14.7–94.7)	4/5, 80.0 (28.4–99.5)
ATN: PPV, *n*/*N*, % (95% CI)	6/8, 75.0 (34.9–96.8)	8/9, 88.9 (51.8–99.7)
ATN: NPV, *n*/*N*, % (95% CI)	3/3, 100 (29.2–100)	4/5, 80.0 (28.4–99.5)
Rejection: TP/FP/FN/TN	3/0/1/7	0/0/2/12
Rejection: Sensitivity, *n*/*N*, % (95% CI)	3/4, 75.0 (19.4–99.4)	0/2, 0 (0–84.2)
Rejection: Specificity, *n*/*N*, % (95% CI)	7/7, 100 (59.0–100)	12/12, 100 (73.5–100)
Rejection: PPV, *n*/*N*, % (95% CI)	3/3, 100 (29.2–100)	not estimable (no positive scans)
Rejection: NPV, *n*/*N*, % (95% CI)	7/8, 87.5 (47.3–99.7)	12/14, 85.7 (57.2–98.2)

ATN = acute tubular necrosis; CI = confidence interval; FN = false negative; FP = false positive; NPV = negative predictive value; PPV = positive predictive value; TN = true negative; TP = true positive.

## Data Availability

The data presented in this study are available on request from the corresponding author because of institutional restrictions on patient-level data.

## Supplementary Materials

The following supporting information can be downloaded at: https://www.mdpi.com/article/10.3390/diagnostics16193193/s1, Table S1. Hilson transplant perfusion index recorded in the original reports, by scintigraphy category and by histological diagnosis. The index was recorded in 33 of 42 studies; one report stated that it could not be calculated and eight reports did not record it. Normal values are below 150. Table S2. Clinical course of the 17 transplants that were not biopsied, by scintigraphy category. These data are descriptive; they were not used as a reference standard and do not enter any ac-curacy calculation. Table S3. Exploratory stratification by timing of scintigraphy and by era of transplantation. Counts only; no inferential comparison performed. Table S4. Comparison of transplants in which renal scintigraphy was performed with those in which it was not, from the center transplant registry, January 2016 to February 2025. The registry contains 483 transplants in this window, of which 73 have a nuclear medicine record; the 42 transplants in the analytic cohort are the subset in which scintigraphy was performed within 90 days for suspected graft dysfunction and complete records were available.
