# Supplementary material for: Diagnostic Performance of 99mTc-MAG3 Renal Scintigraphy for Acute Tubular Necrosis and Acute Rejection in Kidney Transplant Recipients with Suspected Graft Dysfunction During the First 90 Days: A Single-Center, Biopsy-Referenced Retrospective Study
Source: Diagnostics (Basel). 2026 Oct 1;16(19):3193. doi: 10.3390/diagnostics16193193 (PMC13664237; doi:10.3390/diagnostics16193193)
Supplement: Supplementary file 1 [file diagnostics-16-03193-s001.zip › diagnostics-4534706-supplementary.pdf]

diagnostics

MDPI

Supplementary Materials

Table S1. Hilson transplant perfusion index recorded in the original reports, by scintigraphy category and by histological diagnosis. The index was recorded in 33 of 42 studies; one report stated that it could not be calculated and eight reports did not record it. Normal values are below 150.

Group	n with index	Median (IQR)	Range	Index above 150, n (%)
By scintigraphy category				
ATN pattern	24	95 (64–139)	19–521	6 (25)
Rejection pattern	3	185 (129–210)	73–234	2 (67)
Normal	5	31 (16–43)	11–86	0 (0)
Obstruction	1	26	26	0 (0)
By histological diagnosis (biopsied transplants)				
ATN	11	90 (56–198)	32–521	4 (36)
T-cell-mediated rejection	4	59 (41–101)	31–185	1 (25)
Antibody-mediated rejection	1	234	234	1 (100)
Normal histology	2	49 (30–67)	11–86	0 (0)

Table S2. Clinical course of the 17 transplants that were not biopsied, by scintigraphy category. These data are descriptive; they were not used as a reference standard and do not enter any accuracy calculation.

Scintigraphy category	n	Delayed graft function, n/N	eGFR day 30, median (n)	eGFR day 90, median (IQR) (n)	eGFR day 90 of 30 or more, n/N
ATN pattern	14	12/13	35 (12)	45 (25–56) (11)	7/11
Normal	2	0/2	74 (2)	59 (2)	2/2
Obstruction	1	0/1	38 (1)	29 (1)	0/1

eGFR in mL/min/1.73 m^2. For comparison, median eGFR at day 90 in biopsied transplants was 37 with histological ATN (n = 15), 33 with T-cell-mediated rejection (n = 5), 40 with antibody-mediated rejection (n = 1) and 79 with normal histology (n = 4).

Table S3. Exploratory stratification by timing of scintigraphy and by era of transplantation. Counts only; no inferential comparison performed.

Stratum	Transplants, n	Biopsied, n	Scintigraphy categories (ATN / rejection / normal / obstruction)	ATN: TP / FP / FN / TN	Rejection: TP / FP / FN / TN
Scan on days 2 to 14	34	20	28 / 1 / 4 / 1	13 / 3 / 0 / 4	1 / 0 / 3 / 16
Scan on days 15 to 30	6	4	3 / 1 / 2 / 0	1 / 0 / 1 / 2	1 / 0 / 0 / 3
Scan on days 31 to 90	2	1	0 / 1 / 1 / 0	0 / 0 / 0 / 1	1 / 0 / 0 / 0
Transplanted 2016 to 2020	19	14	15 / 0 / 3 / 1	11 / 1 / 1 / 1	0 / 0 / 1 / 13
Transplanted 2021 to 2025	23	11	16 / 3 / 4 / 0	3 / 2 / 0 / 6	3 / 0 / 2 / 6

Table S4. Comparison of transplants in which renal scintigraphy was performed with those in which it was not, from the center transplant registry, January 2016 to February 2025. The registry contains 483 transplants in this window, of which 73 have a nuclear medicine record; the 42 transplants in the analytic cohort are the subset in which scintigraphy was performed within 90 days for suspected graft dysfunction and complete records were available.

Characteristic	Scanned (n = 73)	Not scanned (n = 410)	p value
Age, years, mean (SD)	52.6 (14.9)	51.2 (14.7)	0.47
Male sex, n/N (%)	50/73 (68)	303/410 (74)	0.39
Deceased donor, n/N (%) [a]	21/54 (39)	62/313 (20)	0.004
Retransplantation, n/N (%)	11/73 (15)	46/409 (11)	0.33
Delayed graft function, n/N (%)	38/73 (52)	45/396 (11)	<0.001
Cold ischemia time, minutes, mean (SD)	426 (266) [n = 33]	245 (152) [n = 257]	<0.001
T-cell-mediated rejection recorded, n/N (%)	5/69 (7)	12/393 (3)	0.15
Antibody-mediated rejection recorded, n/N (%)	5/70 (7)	11/391 (3)	0.08

[a] Donor type was not recorded for 110 of 483 transplants (23%), hence the reduced denominators. Referral for scintigraphy tracked the severity of early graft dysfunction, whereas recipient age, sex and retransplantation rate did not differ. SD = standard deviation.

Disclaimer/Publisher's Note: The statements, opinions and data contained in all publications are solely those of the individual author(s) and contributor(s) and not of MDPI and/or the editor(s). MDPI and/or the editor(s) disclaim responsibility for any injury to people or property resulting from any ideas, methods, instructions or products referred to in the content.
